# Observing Third-Party Attentional Relationships Affects Infants' Gaze Following: An Eye-Tracking Study

**DOI:** 10.3389/fpsyg.2016.02065

**Published:** 2017-01-18

**Authors:** Xianwei Meng, Yusuke Uto, Kazuhide Hashiya

**Affiliations:** ^1^Graduate School of Human-Environment Studies, Kyushu UniversityFukuoka, Japan; ^2^Japan Society for the Promotion of ScienceTokyo, Japan; ^3^Faculty of Human-Environment Studies, Kyushu UniversityFukuoka, Japan

**Keywords:** infant, third-party interaction, gaze following, shared attention, early communication

## Abstract

Not only responding to direct social actions toward themselves, infants also pay attention to relevant information from third-party interactions. However, it is unclear whether and how infants recognize the structure of these interactions. The current study aimed to investigate how infants' observation of third-party attentional relationships influence their subsequent gaze following. Nine-month-old, 1-year-old, and 1.5-year-old infants (*N* = 72, 37 girls) observed video clips in which a female actor gazed at one of two toys after she and her partner either silently faced each other (face-to-face condition) or looked in opposite directions (back-to-back condition). An eye tracker was used to record the infants' looking behavior (e.g., looking time, looking frequency). The analyses revealed that younger infants followed the actor's gaze toward the target object in both conditions, but this was not the case for the 1.5-year-old infants in the back-to-back condition. Furthermore, we found that infants' gaze following could be negatively predicted by their expectation of the partner's response to the actor's head turn (i.e., they shift their gaze toward the partner immediately after they realize that the actor's head will turn). These findings suggested that the sensitivity to the difference in knowledge and attentional states in the second year of human life could be extended to third-party interactions, even without any direct involvement in the situation. Additionally, a spontaneous concern with the epistemic gap between self and other, as well as between others, develops by this age. These processes might be considered part of the fundamental basis for human communication.

## Introduction

Coordinated and cooperative social interactions and communication are essential factors of humans. Various types of psychological biases (e.g., the motive to help others; Trivers, 1971; Tomasello et al., 2005; Warneken and Tomasello, 2007) and morphological cues (e.g., the unique morphology of the human eye; Kobayashi and Kohshima, 1997; Kobayashi and Hashiya, 2011) have been argued to be characteristics that make humans socially unique. In terms of ontogeny, the rudiments of social interaction seem to be found from infancy. Research studies in developmental science have revealed that, from early on, human babies show a high sensitivity and response to social stimuli. For instance, newborn infants prefer a self-directed face-like stimulus (Goren et al., 1975; Farroni et al., 2002), and through the first half year of life they come to be sensitive to, and influenced by, direct gaze in social engagement, face recognition, and gaze-following behavior (Haith et al., 1977; Hains and Muir, 1996; Senju and Csibra, 2008). From the second year of infancy, they communicate with others, taking account of their attentional states (i.e., whether the other is perceptually monitoring an event) and knowledge states (i.e., whether the other knows of an event; Saylor and Ganea, 2007; Meng and Hashiya, 2014).

However, it is also important to note that, infants' daily experience with interactions is not limited to ones that they are directly involved in; observation of, and attention to, the interaction between others (such as their parents) also forms an important part of their experience (Nowak and Sigmund, 1998; Hamlin et al., 2007). In fact, monitoring others' interactions has been argued to play a crucial role in early learning (Rogoff et al., 2003). For instance, studies of early language development have demonstrated that infants do learn from third-party conversations and surprisingly, sometimes it may be more efficient than learning in a dyadic context (e.g., learning of pronouns; Oshima-Takane, 1988; Oshima-Takane et al., 1996; also, see the related research into “overheard speech”: Schieffelin and Ochs, 1986; Lieven, 1994; Akhtar, 2005). Several studies have examined infants' understanding of third-party conversations. In these studies, infants' looking behavior while watching face-to-face, and back-to-back conversations was investigated. The results indicated that, from 6 months of age, infants show more gaze shifts between the face-to-face speakers than the back-to-back speakers, in accordance with the flow of the conversation (Augusti et al., 2010). Furthermore, 1-year-old infants show larger pupil dilation for face-to-face conversations (Gustafsson et al., 2016) and they have stronger expectations about the receiver's response in face-to-face contexts when the sender utters a sentence than when she produces non-speech sounds (Thorgrimsson et al., 2015). These findings suggest that, from their first year, infants show particular response (preference in many of the studies) to attend to a typical pattern of conversation and expect face-to-face interaction as a natural form in communication.

Despite the early sensitivity to third-party conversations, it is not well understood how infants recognize and respond to the structure of the third-party interaction itself. As a specific case, for example, let us imagine a situation in which you are observing two individuals who are looking at each other and who subsequently shift their gaze toward an object. We typically assume that the individuals have some common background knowledge that has led to their particular joint action of gazing at the object (Clark and Marshall, 1981). In other words, the specific form of interaction, such as shared attention (e.g., looking at each other), might provide a cue for an observer to interpret others' mental states. In fact, watching others' shared attention has been found to influence adults' gaze-following. Böckler and her colleagues have conducted a series of well-controlled experiments and revealed that, at least in adults with typical development, gaze-following is modulated by the preceding observation of shared attention between others (Böckler et al., 2011, 2014). In their research, the participants observed a scene in which two faces looked at each other, or away from each other, and subsequently jointly shifted gaze to one location. Then, a target appeared either at a cued location (i.e., the location to which the models' faces jointly shifted gaze) or a non-cued location (i.e., the opposite); the participants were requested to detect the target as quickly as possible. The results demonstrated that the gaze cueing effect (i.e., a faster response to objects appearing at the cued location) was found only when participants observed the faces looking at each other (Böckler et al., 2011). This finding suggests that gaze following can be influenced through a top-down process even without a direct communicative intent toward the observer and as with the direct gaze effect, the enhanced processing of others' gaze elicited by observing their shared attention might also play a crucial role in learning (Böckler et al., 2016).

By applying a modified version of the procedure mentioned above, the current study focused on the developmental origin of the recognition of, and response to, others' shared attention—one typical structure of third-party interaction—by examining at what age, and whether/how infants' reflexive gaze following is modulated by observing such structure. Gaze following is found in infants from early on (Scaife and Bruner, 1975; D'Entremont et al., 1997; Hood et al., 1998) and even 6-month-old infants demonstrate a robust tendency to follow others' gaze after receiving an ostensive signal (e.g., being gazed at; Senju and Csibra, 2008). Specifically, infants observed video clips in which a female actor gazed at one of two toys after she and her partner either faced each other (face-to-face condition) or looked in opposite directions (back-to-back condition). The infants' looking behavior for the videos was recorded using an eye tracker, and how they scanned the interactions and whether they followed the actor's gaze was analyzed.

We focused on 9-month-old, 1-year-old, and 1.5-year-old infants in the current study, reflecting the existing evidence, and proposed hypotheses as follows. First, a previous study with a habituation-of-looking-time procedure found that 10-month-old, but not 9-month-old infants, could discriminate between two people in silent movies with mutual versus averted gaze (Beier and Spelke, 2012; Exp. 1). Although a study examining infants' gaze shifts for the face-to-face and back-to-back models suggested that 9-month-old infants were already sensitive to the visual differences between these interaction forms even in static visual images (i.e., they showed more gaze shift frequency for the face-to-face models; Handl et al., 2013), the relationship between the infants' recognition of third-party interactions and the inconsistent responses to these different measures used in these studies remains unclear. Thus, we included both 9-month-old and 1-year-old infants to test whether they perform differently due to their developing understanding of third-party interactions. Second, we included 1.5-year-old infants in the study to test their response to third-party interactions based on their developing social understanding. With the stimuli used in the current study, neither attentional nor knowledge states are expected to be shared between the models in the back-to-back context. This leads to discrepancies in attentional and knowledge states between the actor and her partner in the situation where the actor turns to look at the object in front of her. One previous study has shown that infants in their second year are sensitive to others' attentional and knowledge states and even spontaneously point to indicate a “new” object for the partner (Meng and Hashiya, 2014). Therefore, it was hypothesized that 1.5-year-old infants will have a higher expectation of response by the partner and thus shift their gaze toward her, due to her inaccessibility to the object-gazing situation, and this dislocation of attention from the actor might negatively affect subsequent gaze following (Hypothesis 1). Third, infants' looking frequency, which measures the number of times that they visually attend to the models (but not looking time, which measures the time that infants spend fixating on the models), tends to be higher in a face-to-face condition than in a back-to-back condition (Augusti et al., 2010; Handl et al., 2013). Thus, it was expected that the same tendency will be found in the 9-month-old, 1-year-old, and 1.5-year-old infants (Hypothesis 2).

## Materials and methods

### Ethics statement

All participants were recruited from a database of children (Infant Scientist of Kyushu University) whose parents had volunteered to participate in infant studies at Kyushu University. In accordance with the Declaration of Helsinki, written informed consent was obtained from the children's caregivers before the experiment was conducted. The procedure was approved by the ethics committee of the Faculty of Human-Environment Studies at Kyushu University.

### Participants

The final sample consisted of 72 infants who were separated into three age groups: 24 9-month-old infants (12 girls; *M*_*age*_ = 288 days, *SD* = 7.01, range = 275–301 days), 24 1-year-old infants (11 girls; *M*_*age*_ = 329.3 days, *SD* = 21.1, range = 303–385 days; including 15 10-, 6 11-, 3 12-month-old infants), and 24 1.5-year-old infants (14 girls; *M*_*age*_ = 523.5 days, *SD* = 28.47, range = 485–579 days; including 8 16-, 9 17-, 5 18-, 2 19-month-old infants). An additional seven infants participated but were excluded because they fussed (*N* = 2; 9-months-old), ate confectionary during the experiment (*N* = 1; 1.5-year-old), or did not meet the criteria for analysis, which required available data on at least three trials in the interaction phase (*N* = 4; one 9-month-old, two 1-year-olds, and one 1.5-year-old). All infants were from Japanese families living in the city of Fukuoka.

### Apparatus

The experiment was completed in an open booth with three draped walls in a quiet room located in the Hospital Campus of Kyushu University. The infants were seated on their mothers' laps, ~60 cm from a monitor (23-inch TFT, 300 Hz, 1920 × 1080 pixels) on which the experimental stimuli were presented. The monitor had a built-in remote Tobii TX300 eye-tracking system (Tobii Technology, Danderyd, Sweden). An experimenter who was outside the booth controlled the calibration, presentation of stimuli, and recording of the infants' eye movements using Tobii Studio 3.2.2 (Tobii Technology, Danderyd, Sweden). Two desktop speakers, which were connected to the computer and hidden from view behind the monitor, transmitted electronic sounds to maintain the infants' attention toward the stimuli, as is mentioned in the stimuli section.

### Stimuli

**Figure 1:**
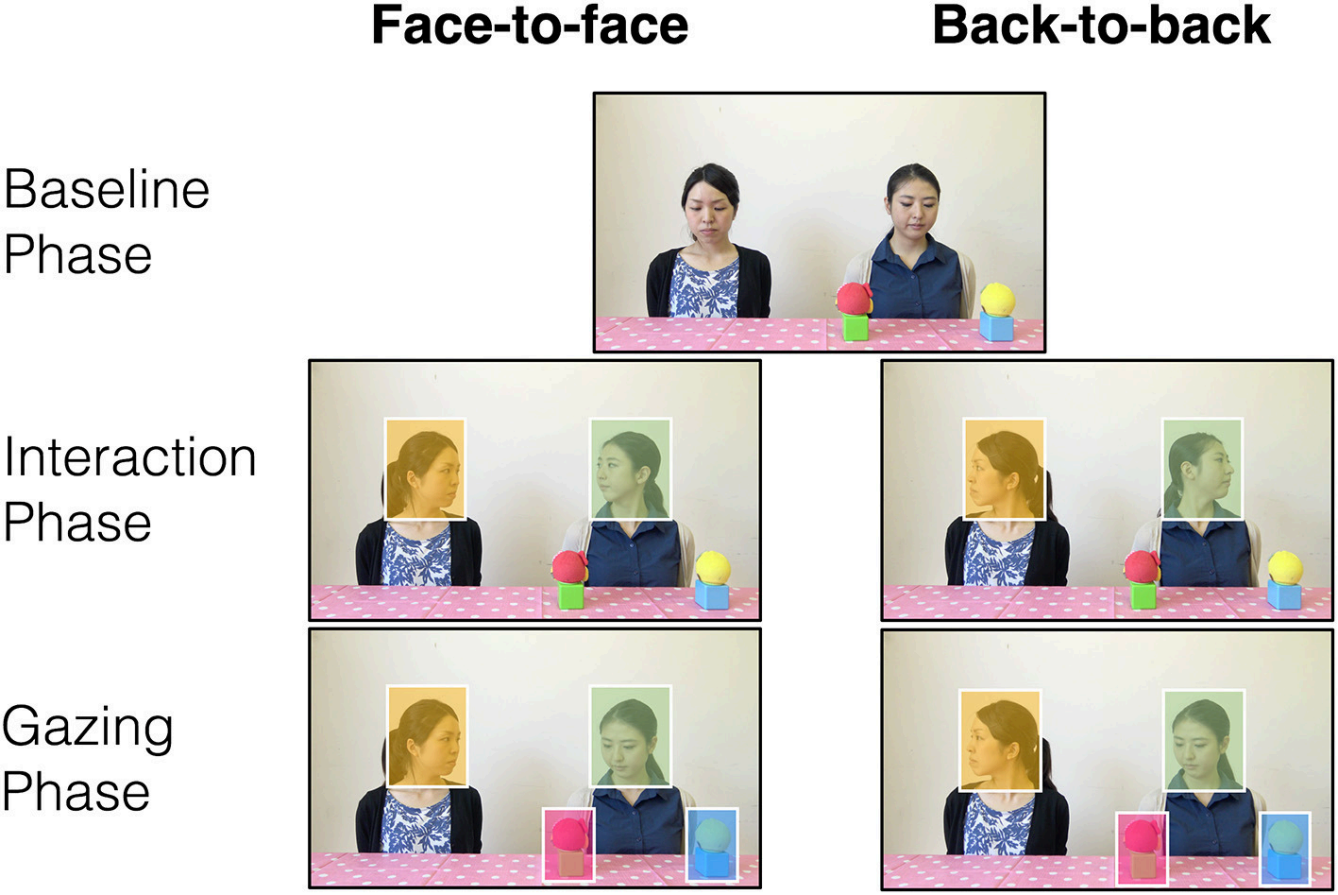
**Samples of the experimental stimuli presented in the face-to-face and back-to-back conditions across the three phases**. Colored rectangular areas indicate the areas of interest for analysis.

Infants were presented with 12 video clips (trials) in total, each with a duration of 11 s. The clips were divided into three phases. The first phase was the *baseline phase*, which started with two female models (i.e., the “actor” and the “partner”) presented on the screen, who were visible from the shoulders up and looking downward (2 s). Two objects (i.e., combinations of a yellow or red sphere on top of a cube) were placed in front of the actor, equidistant from her. The two objects were 30 cm apart. When a short “beep” sound occurred 1.3 s from the beginning, the two models raised their heads while keeping their gaze downward (1 s). This was followed by the *interaction phase*, which differed between conditions. The models looked at each other in the face-to-face condition, or looked away from each other in the back-to-back condition (2 s). The third phase was the *gazing phase*. During this phase, the actor turned her head and looked toward one of the two objects (1 s), and fixated on the object for a further 5 s. In this phase, the partner did not show any movements. During the whole sequence, the models kept a neutral facial expression, were silent, and importantly, they never gazed at the infants (i.e., they never looked into the video camera lens during the recording of the stimuli; Figure 1; Note: Written informed consent was obtained from the actors for publication of the accompanying images).

To ensure that the current design could determine the effect of the models' attentional relationship, we used silent stimuli sequences to avoid the influence of the infants' expectation of a natural conversation (Augusti et al., 2010; Thorgrimsson et al., 2015; Gustafsson et al., 2016). Moreover, because infants may quickly learn the turn-taking in a contingent interaction (Canfield and Haith, 1991), the models acted simultaneously while building their attentional relationship (interaction phase), and the partner never produced a response toward the actor when the actor turned to gaze at the object (gazing phase).

The 12 trials were separated into two blocks. Each block consisted of six trials that all belonged to a specific condition (i.e., either the face-to-face or back-to-back condition). In total, a corpus of 32 video clips was prepared because there were two possible positions for the models to sit in (i.e., the actor could sit on either the left or right side), two possible roles for the models to act (i.e., a specific model could play either the role of the actor or partner), two possible directions for the actor to look in (i.e., looking at the object on either the left or right side), and two possible positions for the objects to be placed (i.e., the yellow object could be placed on either the left or right side). We created two types of video clip sequence for each condition (i.e., face-to-face 1 and 2, and back-to-back 1 and 2). Each type included six video clips that were chosen at random according to the condition. The type presented in each condition was counterbalanced across infants.

To maintain the infants' attention toward the stimuli, the six trials were connected by five 4 s inter-stimulus interval videos. In the inter-stimulus interval videos, an object appeared (i.e., a Daruma doll, bird, banana, horse, or duck) that made a sound with a specific movement. The order of the inter-stimulus interval videos shown between two trials was randomized within blocks.

### Procedure

Each infant completed a warm-up phase to establish a cooperative relationship with the experimenters. The infants, caregivers, and experimenters played with toys that were unrelated to the experimental stimuli in a corner of the same room as the experimental booth. Then, each parent was seated in front of the screen with the infant on her lap. After a 5-point calibration, the infants watched the first block of six trials of one specific condition (i.e., either the face-to-face or back-to-back condition). The infants then had a break for ~5 min during which they could play in the warm-up space. Subsequently, they were presented with the second block of six trials from the other condition. Parents were instructed not to initiate interactions with the infant, and that keeping their eyes closed was desirable for reducing the possible experimental noise.

### Data analysis

To specify the infant's gazing behavior, four rectangular areas of interest (AOIs) were created around the actor's head, partner's head, target object (i.e., the object that the actor gazed at), and distractor object (i.e., the object that the actor did not gaze at; Figure 1). Considering possible errors in gaze estimation, we set the AOIs at 35 pixels wider and higher than the heads and objects (Gredebäck and Melinder, 2010; Thorgrimsson et al., 2015). The final sample was obtained based on the criterion that for each participant, gaze fixation in the AOIs that covered the actor or partner's head in the interaction phase had to be found in at least three trials. This criterion was used because the main purpose of the current study was to investigate the effect of observing models' interactions on subsequent gaze following; thus, being aware of the interactions between models in the interaction phase was considered a prerequisite. The infants' looking behavior was coded and analyzed from the interaction phase (starting at 3 s, with a 2 s duration) and the gazing phase (starting from 5 s, with a 6 s duration). All coding was performed with the Tobii Studio software version 3.2.2 (Tobii Technology, Danderyd, Sweden). The Tobii ClearView Fixation Filter was used to classify gaze fixation within a radius of 35 pixels for at least 100 ms (Salvucci and Goldberg, 2000).

#### Interaction phase

##### Looking time

Data were coded and analyzed for the dependent measure of the total gaze fixation duration within the AOIs that covered the actor's and partner's heads.

##### Looking frequency

Data were also coded and analyzed for the dependent measure of the total looking frequency within the two AOIs. A “look” was defined as the time interval between the first fixation on the active AOI and the end of the last fixation within the same active AOI when there were no fixations outside the AOI (Tobii Studio User Manual, Version 3.2).

The two measures of looking time and looking frequency were employed to confirm whether infants paid visual attention to these 2 s interactions and more importantly, to investigate whether their looking behavior differed when observing the models' face-to-face and back-to-back interactions.

#### Gazing phase

##### Gaze following

We investigated whether the infant looked at the target or distractor object after they observed the actor's head turn (i.e., from 6 s). The Gaze Following Scores (GFSs) were calculated by dividing the number of trials in which the first gaze saccade from the actor's head to the objects was directed to the target object, by the total number of trials with a gaze saccade from the actor's head to either of the two objects. Although the most common coding measure used in the gaze-following paradigm involves coding the infant's gaze shift from the first frame of the model's head turn, note that in the current paradigm the actor always started her head turn from a horizontal gaze posture; therefore, which object the actor intended to gaze at could not be identified until she stopped the head turn (Senju and Csibra, 2008; Szufnarowska et al., 2014). To detect the infant's rapid response to the actor's gaze, only this data reduction was based on the unfiltered data points.

##### Gaze shift toward the partner

It was also investigated whether the infant's first gaze shift from the actor's head was to the partner's head or to either one of the objects after the beginning of the actor's head turn (i.e., from 5 s). This measure was used to address the infant's sensitivity to expectation of the partner's response to the actor's head turn. Each coding began from 200 ms after the first frame of the actor's head turn because infants and adults require around 200 ms to initiate a saccade, as is mentioned in previous research (Canfield et al., 1997). To use an analysis consistent with that employed for gaze following (i.e., from 0 s of the onset), we also coded the data without the 200 ms delay. We found that the consistency between the two types of data was exceedingly high (25% of the data was rescored, *N* = 216, κ = 0.973, *p* < 0.001; unweighted Cohen's Kappa statistic).

The data from the interaction phase were coded automatically using the Tobii Studio software. However, because the data collection in the gazing phase was conditional, only the trials in which the infants shifted their gaze from the AOI that covered the actor's head to the other AOIs were counted. Therefore, the gaze data of the gazing phase were coded using replayed movie clips that were exported with a temporal resolution of 30 frames per second. One quarter of the trials were rescored by a coder who was unaware of the experimental conditions and hypotheses. Inter-rater reliability analysis using the unweighted Cohen's Kappa statistic revealed a high consistency among raters (gaze following: *N* = 216, κ = 0.964, *p* < 0.001; gaze shift toward the partner: *N* = 216, κ = 0.96, *p* < 0.001) (Hallgren, 2012).

### Statistical analysis

Statistical analyses were conducted using R, version 3.2.4 (R Foundation for Statistical Computing, Vienna, Austria). All reported *p* values are two-tailed. A false discovery rate criterion of 5% was used to protect against the effects of multiple testing (Benjamini and Hochberg, 1995).

## Results

### Interaction phase

#### Looking time

Infants looked at the two models' heads for *M* = 1.46 s (*SD* = 0.45) in the face-to-face condition and *M* = 1.47 s (*SD* = 0.45) in the back-to-back condition. Because part of the datasets of the group levels within each condition did not have a normal distribution (i.e., the datasets of the 1.5-years-old group in the face-to-face condition, and 1-year-old and 1.5-years-old group in the back-to-back condition; *ps* < 0.001, Jarque-Bera test) and the group factor had three levels, we applied a generalized linear mixed model (GLMM) with gamma error function and inverse link function to analyze the overall dataset in order to mainly test the effects of condition and sex on the response variable. Then, comparisons within each condition were conducted using the Kruskal-Wallis rank sum test to investigate the effect of age group. Note that although no prediction was established about the effect of sex on each of the response variables in the current study, considering that infants were presented with the interactions of female actors, we included sex as an explanatory variable to confirm that female and male infants did not respond differently due to this specificity of stimuli (the same in the following analysis).

Specifically, the overall GLMM was applied with age (9 months, 1 year, 1.5 years), sex (female, male), and attentional relationship type (face-to-face, back-to-back) as fixed effects and individual differences as a random effect. The results indicated that no factor had a significant effect on looking time. We also tested simpler models by dropping single terms and applying likelihood ratio tests (LRTs; using the lrtest function in R). Neither age (LRT; χ^2^ = 0.39, *df* = 5, *p* = 0.82), sex (LRT; χ^2^ = 1.49, *df* = 6, *p* = 0.22), nor attentional relationship type (LRT; χ^2^ = 0.002, *df* = 6, *p* = 0.96) had a significant effect. Furthermore, a one-way analysis of variance of the ranks revealed no significant difference within the three age groups for looking time in the face-to-face (*p* = 0.89) and back-to-back (*p* = 0.75) conditions. These results showed that the looking time toward the models' heads was not significantly influenced by attentional relationship type or sex. In addition, the infants did not show a developmental change in each condition.

#### Looking frequency

The frequency of looking at either of the two models' heads was *M* = 1.96 (*SD* = 0.48) in the face-to-face condition and *M* = 1.67 (*SD* = 0.52) in the back-to-back condition. The Jarque-Bera test determined that the datasets from each group for each condition were drawn from a normally distributed population (*ps* > 0.44). This ratio scale data was analyzed using a three-way mixed analysis of variance design that included age (9 months, 1 year, 1.5 years) and sex (female, male) as between-subjects factors, and attentional relationship type (face-to-face, back-to-back) as a within-subjects factor. The results revealed a main effect of attentional relationship type, which showed that infants in the face-to-face condition looked more frequently at the two models' heads than those in the back-to-back condition [*F*_(1, 66)_ = 17.48, *p* < 0.001, η_*p*_^2^ = 0.21]. Moreover, the age factor showed a marginal, though not statistically significant, main effect [*F*_(2, 66)_ = 2.96, *p* = 0.059, η_*p*_^2^ = 0.08]. The subsequent multiple comparisons for the age factor showed that there were marginal differences between the 9-month-old (*M* = 1.71, *SD* = 0.44) and 1.5-year-old (*M* = 1.98, *SD* = 0.53) groups (*p* = 0.028, *adj. p* = 0.083), and between the 1-year-old (*M* = 1.75, *SD* = 0.54) and 1.5-year-old groups (*p* = 0.058, *adj. p* = 0.087).

Furthermore, to confirm the reliability of these effects observed for attentional relationship type and the marginally significant age group differences (i.e., 9-months-old vs. 1.5-years-old and 1-year-old vs. 1.5 years-old) for looking frequency, we also fitted a model with a Gaussian error function and identity link function. The overall GLMM was applied with age (9 months, 1 year, 1.5 years), sex (female, male), and attentional relationship type (face-to-face, back-to-back) as fixed effects and individual differences as a random effect. Note that for the age factor, we aimed to focus on the possible differences between the younger two groups (9 months, 1 year) and the older group (1.5 years); thus, the 1.5-years-old group was set as a reference level. In accordance with the analysis of variance results, the GLMM revealed two significant predictors of looking frequency: age (LRT; χ^2^ = 6.32, *df* = 5, *p* = 0.042) and attentional relationship type (LRT; χ^2^ = 16.58, *df* = 6, *p* < 0.001). Specifically, younger infants (9-month-olds: β = −0.27, *t* = −2.33, *p* = 0.023; 1-year-olds: β = −0.23, *t* = −2.02, *p* = 0.048) demonstrated less looking frequency than 1.5-year-olds, and infants in the face-to-face condition (compared to the back-to-back condition) demonstrated more looking frequency toward the models' heads (β = 0.29, *t* = 4.24, *p* < 0.001).

### Gazing phase

#### Gaze following

To test whether infants' gaze following (i.e., the probability of the trial number in which the infants followed the actor's gaze) was influenced by attentional relationship type, sex, or age, we fitted a model with a binomial error function and logit link function (1 = target object was gazed at first, 0 = distractor object was gazed at first). The overall GLMM was applied with age (9 months, 1 year, 1.5 years), sex (female, male), and attentional relationship type (face-to-face, back-to-back) as fixed effects and individual differences as a random effect.

The results showed that neither age (LRT; χ^2^ = 1, *df* = 4, *p* = 0.605), sex (LRT; χ^2^ = 1.93, *df* = 5, *p* = 0.165) nor attentional relationship type (LRT; χ^2^ = 1.42, *df* = 5, *p* = 0.233) had a significant effect. Furthermore, no significant effect of interaction between attentional relationship type and age was found when we fitted the model using this interaction and sex as fixed effects and individual differences as a random effect (LRT; χ^2^ = 2.52, *df* = 3, *p* = 0.774). We also examined the effect of age directly within each attentional relationship type, with age and sex as fixed effects and individual differences as a random effect. No difference was found between each age group in each attentional relationship type (LRT; *ps* > 0.787).

Then, we investigated the main question of whether the GFSs were significantly different from a 0.5 level of chance; that is, whether infants initially gazed at the target object in more or less than half of the trials with a gaze saccade to the objects. The distribution of GFSs from each group for each condition was confirmed as normal with a Jarque-Bera test (*ps* > 0.20), and a two-tailed one-sample *t*-test was employed for the analysis. The results indicated that all but the 1.5-year-old infants in the back-to-back condition [58.5%, *SD* = 0.276, *t*_(23)_ = 1.5, *p* = 0.147, *d* = 0.31, *adj. p* = 0.147] followed the actor's gaze in more than 50% of trials. Specifically, the 9-month-old infants followed the actor's gaze in 66.2% [*SD* = 0.282, *t*_(23)_ = 2.81, *p* = 0.01, *d* = 0.57, *adj. p* = 0.03] and 61.6% of trials [*SD* = 0.213, *t*_(23)_ = 2.67, *p* = 0.014, *d* = 0.54, *adj. p* = 0.028], and the 1-year-old infants followed it in 73.2% [*SD* = 0.248, *t*_(23)_ = 4.59, *p* < 0.001, *d* = 0.94, *adj. p* = 0.001] and 62.2% of trials [*SD* = 0.228, *t*_(23)_ = 2.623, *p* = 0.015, *d* = 0.54, *adj. p* = 0.023] in the face-to-face and back-to-back conditions, respectively. However, the 1.5-year-old infants showed this tendency only in the face-to-face condition, for 63.8% of trials [*SD* = 0.263, *t*_(23)_ = 2.56, *p* = 0.017, *d* = 0.52, *adj. p* = 0.021].

#### Gaze shift toward the partner

Here we tested whether the infants' sensitivity to expectation of the partner's response to the actor's head turn (i.e., shifting their gaze toward the partner immediately after they realized that the actor's head had turned) was influenced by attentional relationship type, sex, or age. The data were analyzed using models with a binomial error function and logit link function (1 = the partner was gazed at first, 0 = objects were gazed at first). The overall GLMM was applied with age (9 months, 1 year, 1.5 years), sex (female, male), and attentional relationship type (face-to-face, back-to-back) as fixed effects and individual differences as a random effect. The analysis indicated a marginal, though not significant, effect of attentional relationship type (LRT; χ^2^ = 3.11, *df* = 5, *p* = 0.078): infants in the back-to-back condition seemed to initially shift gaze to the partner in more trials than those in the face-to-face condition (β = −0.29, *z* = −1.76, *p* = 0.078). However, this was not the case for sex (LRT; χ^2^ = 0.12, *df* = 5, *p* = 0.729).

**Figure 2:**
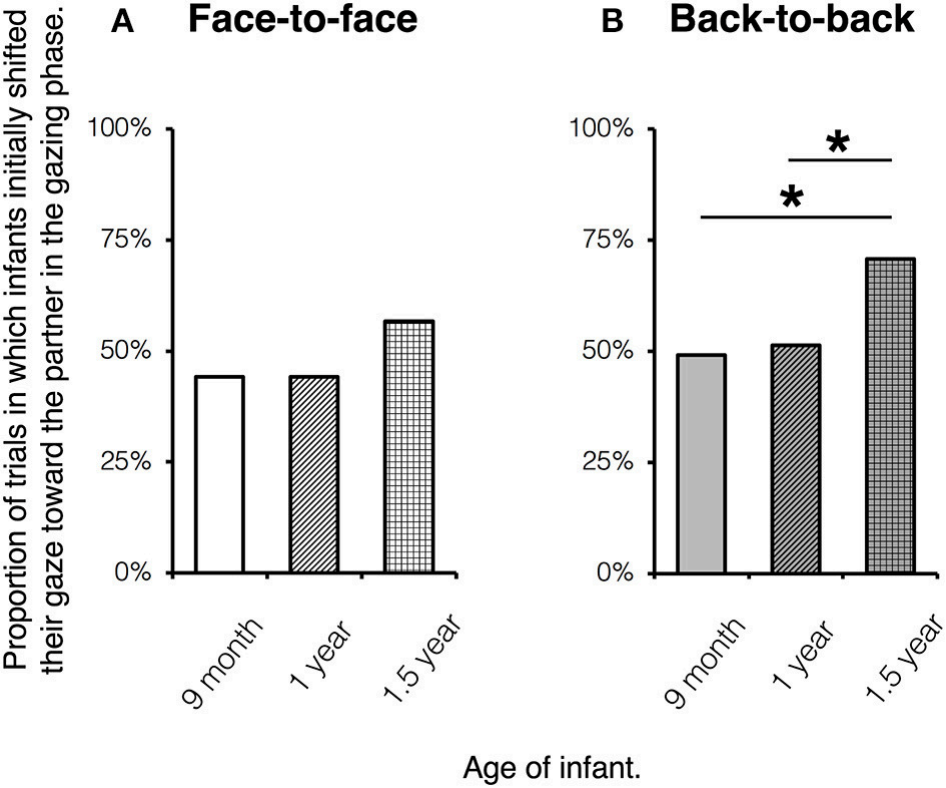
**The results of the gaze shifts toward the partner in the face-to-face (A) and back-to-back (B) conditions**. The proportion was calculated by dividing the number of trials with a first gaze saccade to the partner by the total number of trials with a gaze saccade to either the partner or objects (^*^adj. p < 0.05).

The overall GLMM revealed a significant effect of age (LRT; χ^2^ = 8, *df* = 4, *p* = 0.018), and a significant effect of the interaction between attentional relationship type and age was found when we further fitted the model using the interaction and sex as fixed effects and individual differences as a random effect (LRT; χ^2^ = 11.71, *df* = 3, *p* = 0.039). Therefore, we conducted further comparisons among age groups within each attentional relationship type, with age and sex as fixed effects and individual differences as a random effect. It was found that, in the face-to-face condition, the response variable was not influenced by either age (LRT; *adj. ps* > 0.166) or sex (LRT; *adj. ps* > 0.978). However, in the back-to-back condition, although no effect of sex (LRT; *adj. ps* > 0.645) was found, the response variable was influenced significantly by age. That is, the 1.5-year-olds showed a higher probability to shift their gaze toward the partner than the 1-year-olds (LRT; χ^2^ = 6.6, *df* = 3, *p* = 0.01, *adj. p* = 0.03; β = 0.99, *z* = 2.54, *p* = 0.011, *adj. p* = 0.034) and the 9-month-olds (LRT; χ^2^ = 6, *df* = 3, *p* = 0.014, *adj. p* = 0.021; β = 0.87, *z* = 2.36, *p* = 0.018, *adj. p* = 0.028) (Figure 2).

#### Predicting subsequent gaze following through the gaze shift toward the partner

We examined whether infants initially looking at the partner influenced their subsequent gaze following. First, we set the number of trials in which infants followed the actor's gaze as the response variable, the probability of gaze shifts toward the partner as a fixed effect, and individual differences as a random effect. The model was fitted using the Poisson error function and log link function. We found that the predictor had a strong effect on the response variable. Infants who shifted their gaze toward the partner immediately after the actor starting her head turn followed her gaze to the target object in fewer trials (LRT; χ^2^ = 8.05, *df* = 2, *p* = 0.005; β = −0.486, *z* = −2.84, *p* = 0.005). However, when we set the number of trials in which infants initially gazed at the distractor object as a response variable, the predictor did not show an effect at all (LRT; χ^2^ = 0.01, *df* = 2, *p* = 0.91; β = −0.025, *z* = −0.11, *p* = 0.911). These results indicated that the probability of gaze shifts toward the partner negatively predicted the number of trials in which infants followed the actor's gaze to the target object, but did not predict the number of trials in which infants gazed at the distractor object first.

Second, we directly examined the relationship between the GFSs and the tendency to initially shift gaze toward the partner. A model was fitted with a Gaussian error function and identity link function, including the GFSs as the response variable, the probability of gaze shifts toward the partner as a fixed effect, and the individual differences as a random effect. It was found that the probability of trials with gaze shift toward the partner significantly negatively affected the GFSs (β = −0.158, *t* = −2.35, *p* = 0.022).

## Discussion

The current study aimed to further knowledge about early recognition of third-party interactions by investigating infants' observation of third-party attentional relationships and whether/how they influence the infants' subsequent gaze following. Infants were shown a model (the actor) gazing at one of two toys, following either of two conditions: one in which the actor and partner (appearing in video clips) faced each other, and one in which they looked in opposite directions. The analysis of looking behavior revealed that 9-month-old and 1-year-old infants robustly followed the actor's gaze in both conditions, but this was not the case for 1.5-year-old infants who showed gaze following in significantly more trials only in the face-to-face condition. Moreover, in the back-to-back condition, the 1.5-year-old infants showed a higher tendency (than the younger age groups) to shift their gaze toward the partner after observing the actor's head turn, which might reflect greater attention to the partner's response. In addition, we found that this tendency predicted the degree of gaze following (as proposed in Hypothesis 1).

In the interaction phase, greater looking frequency (but not longer looking time) to the models was found in the face-to-face condition than in the back-to-back condition (as predicted in Hypothesis 2). This face-to-face effect has also been reported in previous studies that used either dynamic or static stimuli (Augusti et al., 2010; Handl et al., 2013; Gustafsson et al., 2016). Another aspect of our results, although not significant, was that the 1.5-year-old infants tended to show a higher looking frequency than the younger age groups. This is also consistent with previous studies that included infant participants from a wider age range of 9–24 months (Handl et al., 2013). Overall, the results of the interaction phase, which showed how infants observed face-to-face and back-to-back third-party interactions, substantially replicated previous findings.

It is important to note that the interaction phase in the current study did not include a turn-taking structure (especially conversation) as information, which contrasts with previous studies (Augusti et al., 2010; Gustafsson et al., 2016). Instead, we used silent movies and the models acted simultaneously. With this manipulation of the stimuli, the current results allowed us to examine the face-to-face effect in a more direct way, independent of the influence of the mere anticipation of responses based on contingency. On this basis, the results suggest that the combination of the configuration of the bodies and the attentional cues of each body (Handl et al., 2013) might function as sufficient cues to elicit the face-to-face effect.

However, in the gazing phase, infants seemed to respond differently to how they did in the interaction phase, which suggests that the response in the gazing phase could not be regarded as a merely perceptual reiteration of the performance (same pattern of scanning the actors' interaction) in the interaction phase. In contrast to the face-to-face effect found in the interaction phase, infants in the gazing phase were even more likely to immediately shift their gaze toward the partner (but not the objects) in the back-to-back than the face-to-face condition. Furthermore, the current results suggest developmental changes in the infants' response in the gazing phase. Specifically, the 1.5-year-old infants in the back-to-back condition were more likely to initially shift their gaze toward the partner than the younger age groups, although this was not the case in the face-to-face condition.

Perception-based accounts do not seem to fit with these independent developmental trajectories in the face-to-face and back-to-back conditions, especially about the 1.5-year-old infants' stronger tendency to initially shift their gaze toward the partner in the back-to-back condition. Previous studies have demonstrated that eye movement control in early infancy (e.g., in 3-month-olds) is already approaching that of adulthood (Haith et al., 1993; Canfield et al., 1997; Fernald et al., 1998). This suggests that the participants in the current study who are not younger than 9 months old would not show any developmental difference in the accuracy or duration of yielding a gaze shift. Also importantly, the current study focused on the infants' gaze shift toward the partner or the objects within the duration of 6 s, the duration long enough for the participants to perform their gaze shifts, on considering their perceptual development.

A more plausible account, from a communicative view, seems to reflect the development in sensitivity to third-party interactions; 1.5-year-old infants might pay more attention to the partner's response based on some level of understanding and expectation of the partner's attentional and/or knowledge states. The situation that appeared in the back-to-back condition might seem rather unnatural or even odd at first glance, but a similar situation occasionally happens in a natural triad or third-party interactions. In the second year of life, infants start to show not only the capacity to interpret others' attentional and knowledge states but also the tendency to reduce discrepancies in knowledge states between the self and others (e.g., they point to provide information for the other person; Saylor and Ganea, 2007; Meng and Hashiya, 2014). The current study extends this view in dyadic settings to triadic settings. In the back-to-back condition, the actor gazed at one object without the partner's attention (i.e., the partner looked in the opposite direction). The dyadic context in previous studies could be summarized as “I know X, but you do not” (Liszkowski et al., 2007; Meng and Hashiya, 2014). The current study expanded the context to one including a discrepancy in knowledge states between the participant, the actor, and her partner, which can be summarized as “I know that ‘A knows X, but B does not’,” at least when we apply a recursive structure to the context. On this basis, the immediate attention to the partner by the 1.5-month-old infants, as compared to their younger peers, suggests that explicit sensitivity to the discrepancy in the knowledge state in the third-party interaction starts to appear around this age.

However, also interestingly, the infants in the current study did not show explicit attempts to inform the partner about the situation (through pointing gestures, for an example; Liszkowski et al., 2007; Meng and Hashiya, 2014), which does not seem to be consistent with studies demonstrating infants' motive to spontaneously help (Warneken and Tomasello, 2006; Tomasello et al., 2007). Particular properties of the experimental stimuli might explain this: the current study used video clips as stimuli to show infants the third-party interactions, which enabled strict control of the agents' motion and timing of interaction. This clearly contrasts with previous studies, which involved infants in a real interaction (Liszkowski et al., 2007; Meng and Hashiya, 2014). Since infants in the first year have already shown different reactions toward video and real presentations (Diener et al., 2008; Dan and Hiraki, 2009), the understanding of the experimental situation that involves video clips might suppress the explicit informing response.

Regarding our question whether infants' gaze following is modulated by observing third-party attentional relationships, the current findings further suggested the process by which third-party observation affected gaze following, through the finding that looking at the partner negatively predicted subsequent gaze following, which was a common tendency beyond the age groups. Previous studies on behavior have revealed an enhanced process of gaze following after observing shared attention (compare to observing back-to-back interaction), and neuroimaging studies have indicated that the fronto-parietal attention network (e.g., left precentral gyrus) is more active when observing back-to-back than face-to-face interactions, suggesting more shifts of spatial attention might be elicited in the back-to-back context (Böckler et al., 2011, 2014, 2016). The current results seem to be in line with these findings, and might further shed a new light on the underlying mechanism for the “ostensive role” (Böckler et al., 2016) of the face-to-face interaction from a third-person's view in the process of gaze following. In other words, the current results might suggest that the sensitivity, or the epistemic vigilance (Sperber et al., 2010) to the difference between self and other, and between others, might be considered in explaining the participants' response, rather than supposing that the combination of perceptual signals in “face-to-face” or “back-to-back” situations simply releases a particular response.

The current results might also expand our understanding of early learning in a third-party context. Studies of early word acquisition have shown that, beyond the dyadic context (e.g., between the infant and mother), infants also actively learn words from third-party conversations. However, a structure of the third-party conversation generally postulates a cooperative relationship between the informants (e.g., in a face-to-face context; Grice, 1975; Oshima-Takane, 1988; Oshima-Takane et al., 1996; Akhtar, 2005). The current study focused on infants' recognition of the structure of the interaction itself, such as shared attention, and revealed a developing sensitivity in the second year of life. Considering that our daily life includes complex social contexts with various forms of interpersonal relationships, such sensitivities might benefit effective acquisition of social information in a third-party context, enabling the selection of superior sources of information using the structure of interactions as a cue. Future research should investigate how these sensitivities influence early learning processes by examining the effect of observing third-party interactions on word learning. For instance, investigating infants' recognition of the name of objects that were labeled by an adult, after he/she interacted with a partner in different contexts.

The possibility of cultural differences in infants' (or children's) sensitivity to third-party interactions should be another focus of future research. In the current stimuli, the actor gazed at one object while the partner did not show any movement, which might emphasize the saliency of the actor's movements while putting the partner in a subsidiary role. Previous studies have shown that Westerners tend to attend to focal objects in a given context, whereas East Asians are more sensitive to contextual information (e.g., the background; Nisbett and Masuda, 2003; Senzaki et al., 2016). It would be worth investigating whether the developmental trajectories of sensitivity to third-party interactions in Japanese infants (i.e., as examined in the current study) might be observed in infants from other cultures.

The current study provided empirical evidence suggesting that infants in their second year of life start to show sensitivity to the attentional and knowledge states between others in a third-party interaction. This finding offers an important step to further our understanding of the developmental origins of the implicit mechanisms underlying daily social communication in humans.

## Author contributions

Conception and design of the study: XM, KH. Analysis and interpretation of data: XM, KH. Collection and assembly of data: XM, YU. Drafting of the article: XM. Critical revision of the article for important intellectual content: XM, KH. Final approval of the article: XM, YU, KH

## Funding

This work was supported by a Grant-in-Aid for the Japan Society for the Promotion of Science Research Fellow (15J02341), a Grant-in-Aid for Scientific Research on Innovative Areas (25114501) “The Evolutionary Origin and Neural Basis of the Empathetic Systems,” and a Grant-in-Aid for Scientific Research (B) (26280049). The funders had no role in the study design, data collection and analysis, decision to publish, or preparation of the manuscript.

### Conflict of interest statement

The authors declare that the research was conducted in the absence of any commercial or financial relationships that could be construed as a potential conflict of interest.

## Abbreviations

AOIs: areas of interest
GFSs: gaze following scores
GLMM: generalized linear mixed model
LRT: likelihood ratio test.

## References

[B1] Akhtar N. (2005). The robustness of learning through overhearing. Dev. Sci. 8, 199–209. 10.1111/j.1467-7687.2005.00406.x 15720377

[B2] Augusti E. M. Melinder A. Gredebäck G. (2010). Look who's talking: pre-verbal infants' perception of face-to-face and back-to-back social interactions. Front. Psychol. 1:161. 10.3389/fpsyg.2010.00161 21833226 PMC3153775

[B3] Beier J. S. Spelke E. S. (2012). Infants' developing understanding of social gaze. Child Dev. 83, 486–496. 10.1111/j.1467-8624.2011.01702.x 22224547 PMC3686118

[B4] Benjamini Y. Hochberg Y. (1995). Controlling the false discovery rate: a practical and powerful approach to multiple testing. J. R. Stat. Soc. Ser. B 57, 289–300.

[B5] Böckler A. Eskenazi T. Sebanz N. Rueschemeyer S. A. (2016). (How) observed eye-contact modulates gaze following. An fMRI study. Cogn. Neurosci. 7, 55–66. 10.1080/17588928.2015.1053442 25996424

[B6] Böckler A. Knoblich G. Sebanz N. (2011). Observing shared attention modulates gaze following. Cognition 120, 292–298. 10.1016/j.cognition.2011.05.002 21621753

[B7] Böckler A. Timmermans B. Sebanz N. Vogeley K. Schilbach L. (2014). Effects of observing eye contact on gaze following in high-functioning Autism. J. Autism Dev. Disord. 44, 1651–1658. 10.1007/s10803-014-2038-5 24442835

[B8] Canfield R. L. Haith M. M. (1991). Young infants' visual expectations for symmetric and asymmetric stimulus sequences. Dev. Psychol. 27, 198–208. 10.1037/0012-1649.27.2.198

[B9] Canfield R. L. Smith E. G. Brezsnyak M. P. Snow K. L. Aslin R. N. Haith M. M.. (1997). Information processing through the first year of life: a longitudinal study using the visual expectation paradigm. Monogr. Soc. Res. Child Dev. 62, 1–5. 10.2307/1166196 9353949

[B10] Clark H. H. Marshall C. R. (1981). Definite reference and mutual knowledge, in Elements of Discourse Understanding, eds Joshi A. K. Webber B. L Sag I. A. (Cambridge: Cambridge University Press), 10–63.

[B11] Dan H. Hiraki K. (2009). Understanding of television in infants and toddlers. Hum. Dev. Res. CODER Annu. Rep. 23, 115–130. (in Japanese).

[B12] D'Entremont B. Hains S. M. J. Muir D. W. (1997). A demonstration of gaze following in 3-to 6-month-olds. Infant Behav. Dev. 20, 569–572. 10.1016/S0163-6383(97)90048-5

[B13] Diener M. L. Pierroutsakos S. L. Troseth G. L. Roberts A. (2008). Video versus reality: infants' attention and affective responses to video and live presentations. Media Psychol. 11, 418–441. 10.1080/15213260802103003

[B14] Farroni T. Csibra G. Simion F. Johnson M. H. (2002). Eye contact detection in humans from birth. Proc. Natl. Acad. Sci. U.S.A. 99, 9602–9605. 10.1073/pnas.152159999 12082186 PMC123187

[B15] Fernald A. Pinto J. P. Swingley D. Weinbergy A. McRoberts G. W. (1998). Rapid gains in speed of verbal processing by infants in the 2nd year. Psychol. Sci. 9, 228–231. 10.1111/1467-9280.00044

[B16] Goren C. C. Sarty M. Wu P. Y. (1975). Visual following and pattern discrimination of face-like stimuli by newborn infants. Pediatrics 56, 544–549. 1165958

[B17] Gredebäck G. Melinder A. (2010). Infants' understanding of everyday social interactions: a dual process account. Cognition 114, 197–206. 10.1016/j.cognition.2009.09.004 19800056

[B18] Grice H. P. (1975). Logic and conversation, in Syntax and Semantics, 3: Speech Acts, eds Cole P. Morgan J. (New York, NY: Academic Press), 41–58.

[B19] Gustafsson E. Brisson J. Mailloux D. Mainville M. Beaulieu C. Sirois S. (2016). Do infants recognize engagement in social interactions? The Case of Face-to-Face Conversation. Infancy 21, 685–696. 10.1111/infa.12135

[B20] Hains S. M. Muir D. W. (1996). Infant sensitivity to adult eye direction. Child Dev. 67, 1940–1951. 10.2307/1131602 9022223

[B21] Haith M. M. Bergman T. Moore M. J. (1977). Eye contact and face scanning in early infancy. Science 198, 853–855. 10.1126/science.918670 918670

[B22] Haith M. M. Wentworth N. Canfield R. L. (1993). The formation of expectations in early infancy. Adv. Infancy Res. 8, 251–297

[B23] Hallgren K. A. (2012). Computing inter-rater reliability for observational data: an overview and tutorial. Tutor Quant. Methods Psychol. 8:23. 10.20982/tqmp.08.1.p023 22833776 PMC3402032

[B24] Hamlin J. K. Wynn K. Bloom P. (2007). Social evaluation by preverbal infants. Nature 450, 557–559. 10.1038/nature06288 18033298

[B25] Handl A. Mahlberg T. Norling S. Gredebäck G. (2013). Facing still faces: what visual cues affect infants' observations of others? Infant Behav. Dev. 36, 583–586. 10.1016/j.infbeh.2013.06.001 23831615

[B26] Hood B. M. Willen J. D. Driver J. (1998). Adult's eyes trigger shifts of visual attention in human infants. Psychol. Sci. 9, 131–134. 10.1111/1467-9280.00024

[B27] Kobayashi H. Hashiya K. (2011). The gaze that grooms: contribution of social factors to the evolution of primate eye morphology. Evol. Hum. Behav. 32, 157–165. 10.1016/j.evolhumbehav.2010.08.003

[B28] Kobayashi H. Kohshima S. (1997). Unique morphology of the human eye. Nature 387, 767–768. 10.1038/42842 9194557

[B29] Lieven E. V. M. (1994). Crosslinguistic and crosscultural aspects of language addressed to children, in Input and Interaction in Language Acquisition, eds Gallaway C. Richards B. J. (New York, NY: Cambridge University Press), 56–73.

[B30] Liszkowski U. Carpenter M. Tomasello M. (2007). Pointing out new news, old news, and absent referents at 12 months of age. Dev. Sci. 10, F1–F7. 10.1111/j.1467-7687.2006.00552.x 17286836

[B31] Meng X. Hashiya K. (2014). Pointing behavior in infants reflects the communication partner's attentional and knowledge states: a possible case of spontaneous informing. PLoS ONE 9:e107579. 10.1371/journal.pone.0107579 25211279 PMC4161458

[B32] Nisbett R. E. Masuda T. (2003). Culture and point of view. Proc. Natl. Acad. Sci. U.S.A. 100, 11163–11170. 10.1073/pnas.1934527100 12960375 PMC196945

[B33] Nowak M. A. Sigmund K. (1998). Evolution of indirect reciprocity by image scoring. Nature 393, 573–577. 10.1038/31225 9634232

[B34] Oshima-Takane Y. Goodz E. Derevensky J. L. (1996). Birth order effects on early language development: do secondborn children learn from overheard speech? Child Dev. 67, 621–634. 10.2307/1131836

[B35] Oshima-Takane Y. (1988). Children learn from speech not addressed to them: the case of personal pronouns. J Child Lang. 15, 95–108. 10.1017/S0305000900012071 3350879

[B36] Rogoff B. Paradise R. Arauz R. M. Correa-Chávez M. Angelillo C. (2003). Firsthand learning through intent participation. Annu. Rev. Psychol. 54, 175–203. 10.1146/annurev.psych.54.101601.145118 12499516

[B37] Salvucci D. D. Goldberg J. H. (2000). Identifying fixations and saccades in eye-tracking protocols, in Proceedings of the 2000 Symposium on Eye Tracking Research and Applications (Palm Beach Gardens, FL: ACM), 71–78.

[B38] Saylor M. M. Ganea P. (2007). Infants interpret ambiguous requests for absent objects. Dev Psychol. 43, 696–704. 10.1037/0012-1649.43.3.696 17484581

[B39] Scaife M. Bruner J. S. (1975). The capacity for joint visual attention in the infant. Nature 253, 265–266. 10.1038/253265a0 1113842

[B40] Schieffelin B. B. Ochs E. (1986). Language socialization. Annu. Rev. Anthropol. 15, 163–191. 10.1146/annurev.an.15.100186.001115

[B41] Senju A. Csibra G. (2008). Gaze following in human infants depends on communicative signals. Curr. Biol. 18, 668–671. 10.1016/j.cub.2008.03.059 18439827

[B42] Senzaki S. Masuda T. Takada A. Okada H. (2016). The communication of culturally dominant modes of attention from parents to children: a comparison of Canadian and Japanese parent-child conversations during a joint scene description task. PLOS ONE 11:e0147199. 10.1371/journal.pone.0147199 26824241 PMC4733050

[B43] Sperber D. Clément F. Heintz C. Mascaro O. Mercier H. Origgi G. (2010). Epistemic vigilance. Mind Lang. 25, 359–393. 10.1111/j.1468-0017.2010.01394.x

[B44] Szufnarowska J. Rohlfing K. J. Fawcett C. Gredebäck G. (2014). Is ostension any more than attention?. Sci. Rep. 4:5304. 10.1038/srep05304 24931735 PMC4058873

[B45] Thorgrimsson G. B. Fawcett C. Liszkowski U. (2015). 1-and 2-year-olds' expectations about third-party communicative actions. Infant Behav. Dev. 39, 53–66. 10.1016/j.infbeh.2015.02.002 25766104

[B46] Tomasello M. Carpenter M. Call J. Behne T. Moll H. (2005). Understanding and sharing intentions: the origins of cultural cognition. Behav. Brain. Sci. 28, 675–691. 10.1017/S0140525X05000129 16262930

[B47] Tomasello M. Carpenter M. Liszkowski U. (2007). A new look at infant pointing. Child Dev. 78, 705–722. 10.1111/j.1467-8624.2007.01025.x 17516997

[B48] Trivers R. L. (1971). The evolution of reciprocal altruism. Q. Rev. Biol. 35–57. 10.1086/40675523717479

[B49] Warneken F. Tomasello M. (2007). Helping and cooperation at 14 months of age. Infancy 11, 271–294. 10.1111/j.1532-7078.2007.tb00227.x 33412734

[B50] Warneken F. Tomasello M. (2006). Altruistic helping in human infants and young chimpanzees. Science 311, 1301–1303. 10.1126/science.1121448 16513986

